# Simultaneous surgical management of a giant tuberculum sellae meningioma and pregnancy-related complications: a case report and literature review

**DOI:** 10.3389/fonc.2025.1576797

**Published:** 2025-06-04

**Authors:** Muratbek A. Tleubergenov, Nurzhan A. Ryskeldiyev, Dauren S. Baymukhanov, Daniyar K. Zhamoldin, Serik Akshulakov, Aidos Doskaliyev

**Affiliations:** ^1^ Department of Neurosurgery for Brain Pathology, Joint-Stock Company (JSC) “National Center for Neurosurgery, Astana, Kazakhstan; ^2^ Joint-Stock Company (JSC) “National Center for Neurosurgery”, Astana, Kazakhstan

**Keywords:** meningioma, oncology, tumor resection, pregnancy, caesarean section, simultaneous surgery meningioma, simultaneous surgery

## Abstract

Meningiomas, tumors arising from the brain and spinal cord membranes, pose a unique challenge when diagnosed during pregnancy. Their growth can accelerate due to hormonal and hemodynamic changes, necessitating careful clinical evaluation to balance maternal and fetal risks. This article presents a case report and literature review on the successful simultaneous management of a giant tuberculum sellae meningioma and pregnancy-related complications in a 35-year-old woman at 38 weeks of gestation. The patient experienced progressive visual deterioration, prompting an emergency surgical intervention. A multidisciplinary team performed a simultaneous pterional craniotomy with microsurgical tumor resection alongside a cesarean section, ensuring optimal outcomes for both mother and child. Postoperative results demonstrated significant improvement in the patient’s vision and neurological function, with a healthy neonate delivered without complications. The histopathological examination confirmed an atypical meningioma (WHO Grade II), reinforcing the need for long-term follow-up and oncological assessment. In addition to the case report, this study reviews the existing literature on meningiomas during pregnancy, focusing on hormonal influences, diagnostic challenges, and treatment strategies. We discuss the role of multimodal imaging, including MRI without contrast, as a primary diagnostic tool and evaluate the risks associated with different treatment approaches. The review highlights the importance of timely surgical intervention, particularly in cases of rapidly growing or symptomatic tumors, and underscores the feasibility of simultaneous neurosurgical and obstetric procedures when clinically indicated. This review emphasizes the necessity of a multidisciplinary approach involving neurosurgeons, obstetricians, anesthesiologists, and neonatologists to optimize both maternal and fetal outcomes. By combining clinical expertise with a thorough literature analysis, we provide valuable insights into the management of intracranial tumors in pregnancy, contributing to the development of treatment protocols for such complex cases.

## Introduction

Meningioma is a tumor originating from the meninges, the protective layers of tissue (dura mater, arachnoid mater, and pia mater) that encase the brain and spinal cord. It is the most common primary brain tumor in adults and comprise nearly 36% of all intracranial tumors (1). Meningiomas occur more frequently in women than in men, with a ratio of approximately 2:1. Around 20% of all meningiomas are classified as aggressive forms, such as atypical and anaplastic meningiomas representing WHO Grade 2 and WHO Grade 3, respectively (2).

Pregnancy has a distinct impact on the clinical course of meningiomas, although these tumors are exceedingly rare in pregnancy, with a frequency of 2.6 cases per 100,000 pregnancies (3). During gestation, the growth of meningiomas can accelerate significantly due to hormonal and hemodynamic changes. Elevated levels of progesterone and estrogen in the second and third trimesters render tumors expressing progesterone receptors particularly sensitive to these hormones. As a result, meningioma growth occurs in 30-50% of pregnant patients, often accompanied by worsening symptoms, necessitating an individualized approach to diagnosis and treatment. Variability of growth rate and clinical presentation necessiates an individualized approach to diagnosis and treatment (4).

Diagnosis is complicated by the similarity of symptoms, such as headaches, vision disturbances, and seizures, to common manifestations of pregnancy, which can delay identification of the tumor (5). Treatment strategies depend on the tumor’s location, size, growth rate and the patient’s condition, and the stage of pregnancy. In stable cases, observation may be sufficient. However, serious sequelae, including neurological deficits, may require surgical intervention, especially in cases of significant brain edema (6).

Managing these rare cases warrants multidisciplinary input from neurosurgeons, gynecologists, anesthesiologists, and neonatologists. In our study, we present a case of successful treatment of a pregnant patient with a giant meningioma, involving simultaneous neurosurgical resection and cesarean delivery. This case emphasizes the importance of a multidisciplinary approach and ensuring the safety of both the mother and the fetus.

## Case description

We report the case of a 35-year-old woman at 38 weeks of gestation who was admitted to the National Center for Neurosurgery, Astana, Kazakhstan, for a combined surgical procedure involving cesarean section and resection of a giant tuberculum sellae meningioma. The onset of symptoms occurred in July 2024, when the patient developed progressive visual disturbances in the right eye, described as blurring, discomfort, and a “shadow” in the visual field. She was initially evaluated by an ophthalmologist and subsequently referred for neurological assessment. Brain MRI revealed a large extra-axial mass in the tuberculum sellae region with bilateral frontal extension, consistent with a giant meningioma. At 36 weeks of gestation, she was first evaluated by a neurosurgeon; given her stable neurological condition at that time, a conservative management strategy with close monitoring was recommended (**Table 1**). The patient was referred for obstetric consultation to determine the optimal timing and mode of delivery, with a plan for elective cesarean section at full term. Additionally, it was recommended to perform postpartum neuroimaging (control CT and MRI of the brain) to reassess the lesion. To manage potential peritumoral edema, the patient was started on dexamethasone 8 mg twice daily, along with omeprazole 20 mg twice daily for gastroprotection. However, due to subsequent deterioration in visual function, the patient was admitted at 38 weeks in a delayed emergency setting for a planned combined surgical intervention.

**Table 1:** Patient management timeline.

Date	Clinical Event
July 2024	Initial presentation to ophthalmologist with complaints of visual disturbance.
July 13, 2024	Brain MRI performed, revealing a large tuberculum sellae mass.
September 2, 2024	First neurosurgical consultation; neurological status deemed stable.
September 18, 2024 (Morning)	Re-evaluation by neurosurgery and obstetrics teams due to visual deterioration.
September 19, 2024	Emergency simultaneous cesarean section and neurosurgical tumor resection performed.
September 20, 2024	Postoperative ophthalmologic assessment confirming visual stabilization.
September 23, 2024	Obstetric follow-up; no maternal or neonatal complications identified.
September 26, 2024	Patient discharged in stable neurological and general condition.
October 2024	One-month follow-up visit with postoperative brain MRI confirming stable outcome.

### Investigation

At the time of admission on September 18, 2024, the patient was 38 weeks pregnant and complained of worsening bilateral visual acuity, with light perception only, complete loss of peripheral vision in the right eye, and fatigue. Neurological examination revealed a clear sensorium with a Glasgow Coma Scale score of 15/15. No focal motor deficits were present; motor strength was preserved in all extremities (5/5). However, ophthalmological assessment confirmed near-total optic nerve atrophy on the right and partial atrophy on the left, along with temporal hemianopsia and amblyopia.

**Figure 1:**
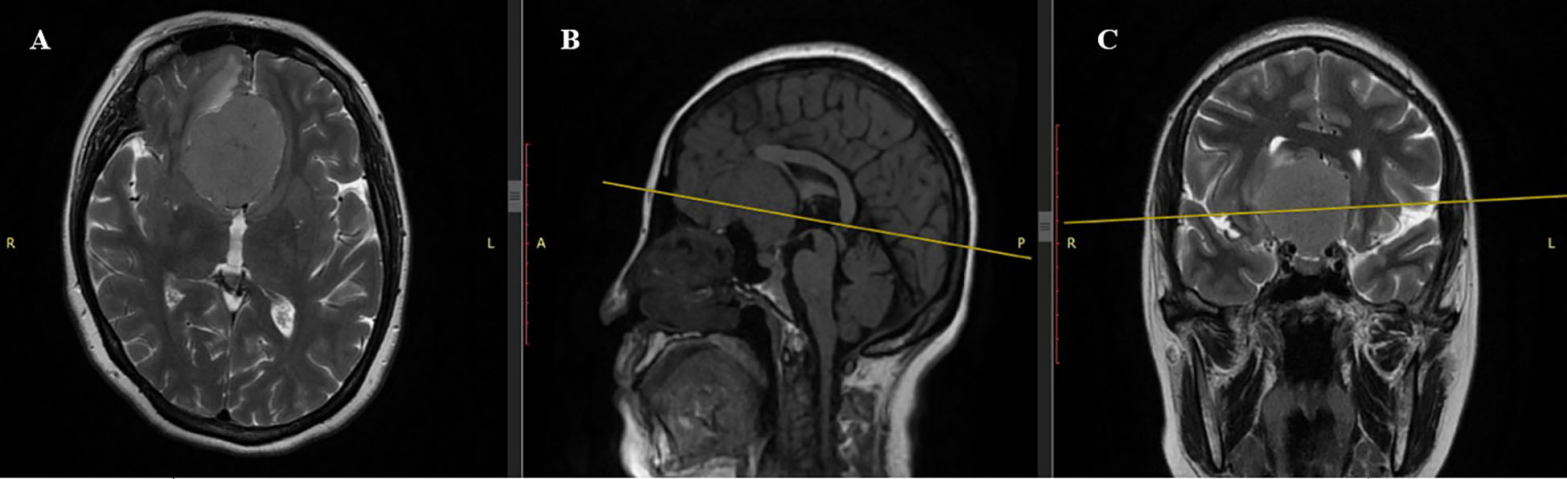
Preoperative MRI scan. **(A)** Axial T2-weighted sequence: Giant tuberculum sellae meningioma (4.81×4.85×4.26 cm) with well-demarcated margins and pronounced isointense signal, causing significant frontal lobe compression. **(B)** Sagittal T1-weighted sequence: Iso- to hypointense lesion with mass effect on basal structures, including the optic chiasm. **(C)** Coronal T2-weighted sequence: Isointense lesion occupying the olfactory groove, minimal peritumoral edema, and displacement of frontal lobes and optic chiasm.

Magnetic resonance imaging (MRI) of the brain revealed a large, well-circumscribed extra-axial mass located at the tuberculum sellae, measuring 4.81 × 4.85 × 4.26 cm (**Figure 1**). The lesion demonstrated isointense signal to gray matter on both T1- and T2-weighted imaging. MRI was performed without contrast administration due to pregnancy, and therefore no assessment of contrast enhancement was made. The mass caused significant compression of the optic chiasm and optic nerves, consistent with the patient’s visual symptoms. There was no evidence of peritumoral edema, hydrocephalus, or hemorrhage. Routine laboratory and biochemical tests, including coagulation profile and urinalysis, were within acceptable limits for surgery. Obstetric evaluation confirmed a singleton pregnancy at full term, with no contraindications for cesarean delivery. The patient had a history of three prior cesarean sections, mild gestational anemia, and gestational diabetes.

### Differential diagnosis

The differential diagnosis in this pregnant patient presenting with progressive bilateral visual impairment, persistent headache, and optic nerve atrophy encompassed several intracranial pathologies, each evaluated in light of specific neuroimaging characteristics. Pituitary macroadenoma was considered due to its frequency during pregnancy and sellar location; however, the lesion did not arise from the pituitary gland, showed no “snowman” or “dumbbell” configuration, and lacked sellar floor remodeling or homogeneous post-contrast enhancement typical of macroadenomas. Craniopharyngioma was excluded based on the absence of hallmark imaging features such as mixed cystic and solid components, calcifications (commonly seen on T1- and susceptibility-weighted images), and no suprasellar extension with third ventricular involvement. Optic nerve glioma, though a consideration due to bilateral visual symptoms, was ruled out because the lesion was clearly extra-axial, dural-based, and compressive rather than infiltrative, and it spared the optic nerves and chiasm parenchyma - unlike the fusiform enlargement and intrinsic T2 hyperintensity typical of gliomas. Metastatic lesions were excluded as there were no multiple enhancing lesions, hemorrhagic components, or ring enhancement on post-contrast T1-weighted images - features commonly associated with brain metastases. Finally, meningioma of the tuberculum sellae was confirmed due to the presence of a well-defined, extra-axial, dural-based mass with broad-based dural attachment, homogeneous enhancement, displacement of the optic chiasm, and typical location along the anterior cranial base. These findings, combined with the rapid clinical progression and histopathological confirmation, supported the final diagnosis.

### Management and treatment

Given the urgent risk of irreversible vision loss and potential fetal compromise due to maternal decompensation, immediate surgical intervention was indicated. To minimize anesthesia-related risks and avoid staged procedures, a single-session, dual-surgery approach was selected. The patient underwent a simultaneous cesarean section and neurosurgical tumor resection. The neurosurgical component involved a right-sided modified pterional craniotomy with microsurgical tumor removal using neuronavigation and high-magnification microscopy. The cesarean section was performed first. A healthy full-term neonate was delivered with Apgar scores of 8–9 at 1 and 5 minutes, respectively, and transferred to the neonatal intensive care unit. Then the patient was positioned supine with the head turned to the left and secured in a rigid three-point fixation system. After antiseptic preparation, a left-sided frontotemporal skin incision (~12–13 cm) was made. A skin-muscle flap was mobilized anteriorly, followed by pericranial dissection. Craniotomy was performed using burr holes and a high-speed drill, yielding a bone flap measuring approximately 8.5 × 7.7 cm. Additional resection of the lateral sphenoid wing and temporal squama was carried out to enhance access to the anterior cranial base. Upon dural opening, the underlying brain appeared moderately tense, with local thickening of the dura. Microsurgical dissection was performed under high magnification with continuous neuronavigation assistance. A solid extra-axial tumor was visualized in the region of the tuberculum sellae, exhibiting a pale-gray color, firm elastic consistency, hypervascularization, and extensive adherence to adjacent structures, including the frontal and temporal lobes, anterior cerebral arteries, and the right optic nerve. Internal decompression was performed using microsurgical instruments with bipolar coagulation. Due to tight adherence to branches of the ACA (anterior cerebral artery) and MCA (middle cerebral artery), subtotal resection of the main tumor bulk (~7 cm) was performed to prevent vascular injury. Capsular remnants were intentionally left in high-risk areas. Brain pulsation improved notably following decompression. Hemostasis was achieved using bipolar cautery and hemostatic agents. The dura was closed with local autologous tissue. The bone flap was partially reattached in a decompressive fashion using interrupted sutures, and a subgaleal (epicranial) drain was subsequently placed beneath the galea aponeurotica. The surgical wound was closed in layers with an intradermal suture.

Anesthesia management involved induction with intravenous propofol and suxamethonium, followed by maintenance with continuous propofol infusion and fentanyl-based opioid analgesia. Controlled mechanical ventilation was adjusted to optimize brain relaxation. Hypotensive anesthesia was employed to minimize intraoperative bleeding, with mean arterial pressure maintained between 65–75 mmHg. Total blood loss was approximately 1000 mL, and intraoperative autotransfusion was performed using cell salvage, recovering 1250 mL of washed red blood cells. The patient received crystalloids, 15% mannitol, dexamethasone, prophylactic antibiotics, and oxytocin.

### Histopathological findings

**Figure 2:**
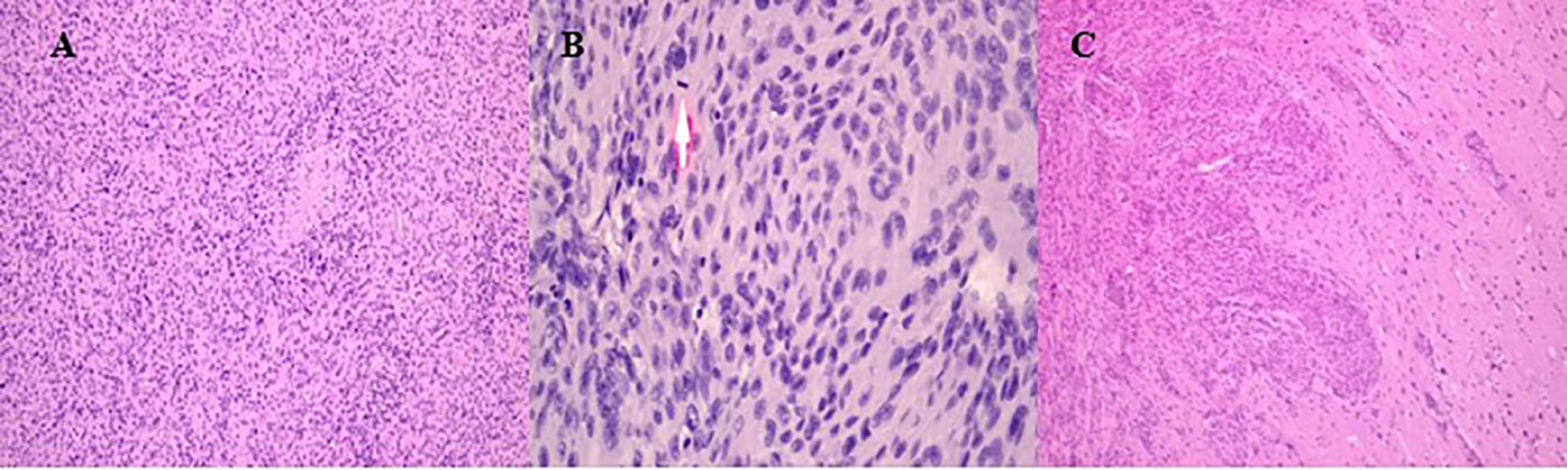
Histopathological results of intraoperative material. **(A)** Tumor fragments showing uniform, round to oval endothelial-like cells arranged in a mosaic-like pattern. **(B)** Mitotic activity with more than 4 figures observed in 10 high-power fields. The arrow shows the figures of mitosis. **(C)** Tumor invasion into adjacent brain parenchyma with necrotic foci and scattered lymphoid infiltrates.

The histopathological analysis revealed a morphological picture consistent with atypical meningioma (WHO Grade 2). Microscopically, tumor fragments consisted of relatively uniform, round, and oval endothelial-like cells of medium size. The cells were arranged in a mosaic-like pattern, closely adhering to each other without clear boundaries. Nuclei were round to oval, occasionally polymorphic. Nucleoli were small and indistinct. Mitotic figures were visible in nuclei (more than 4 figures in 10 high-power fields). The tumor cells formed alveolar, macro-, and micro-concentric structures. Delicate fibrous tissue fibers were observed between the cells. Scattered lymphoid infiltrates, necrotic foci, and areas of tumor invasion into the adjacent brain parenchyma were also noted (**Figure 2**).

### Postoperative course and outcome

**Figure 3:**
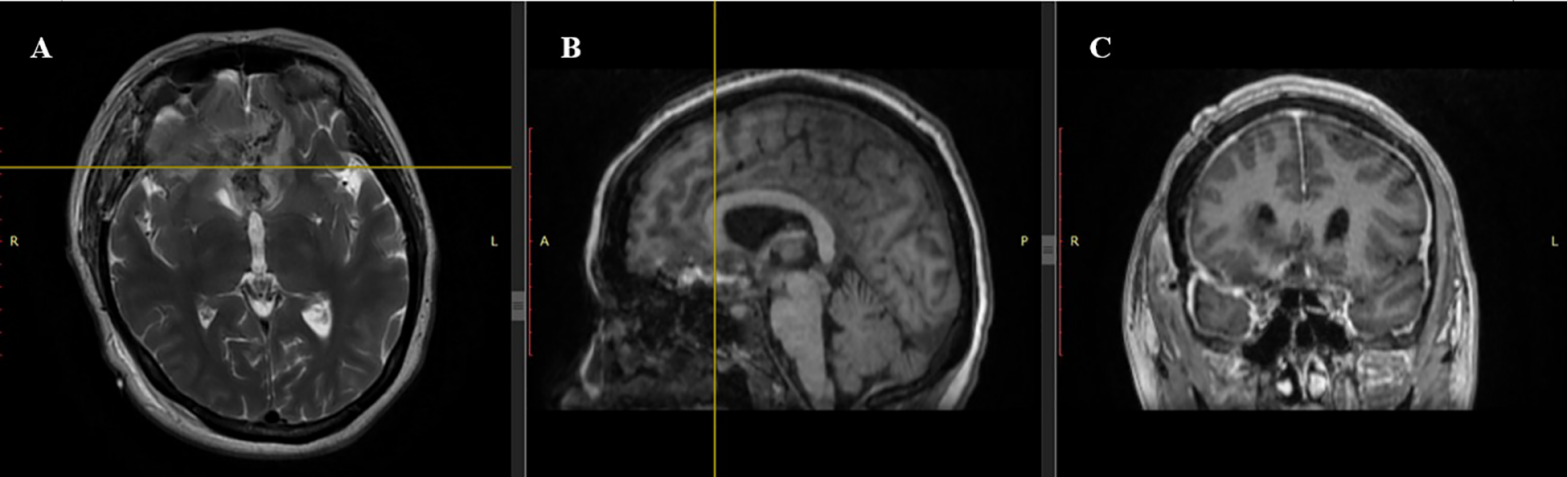
Postoperative MRI scan. **(A)** Axial T2-weighted sequence: Complete resection of the tuberculum sellae meningioma with restored anatomy of the frontal lobes. Minimal postoperative changes are observed without significant edema. **(B)** Sagittal T1-weighted sequence: Hypointense resection cavity with no visible residual tumor. Midline structures are realigned, reflecting resolution of preoperative displacement. **(C)** Coronal T1-weighted sequence: Symmetry of frontal lobes restored with no residual tumor, hematoma, or cerebrospinal fluid accumulation.

**Figure 4:**
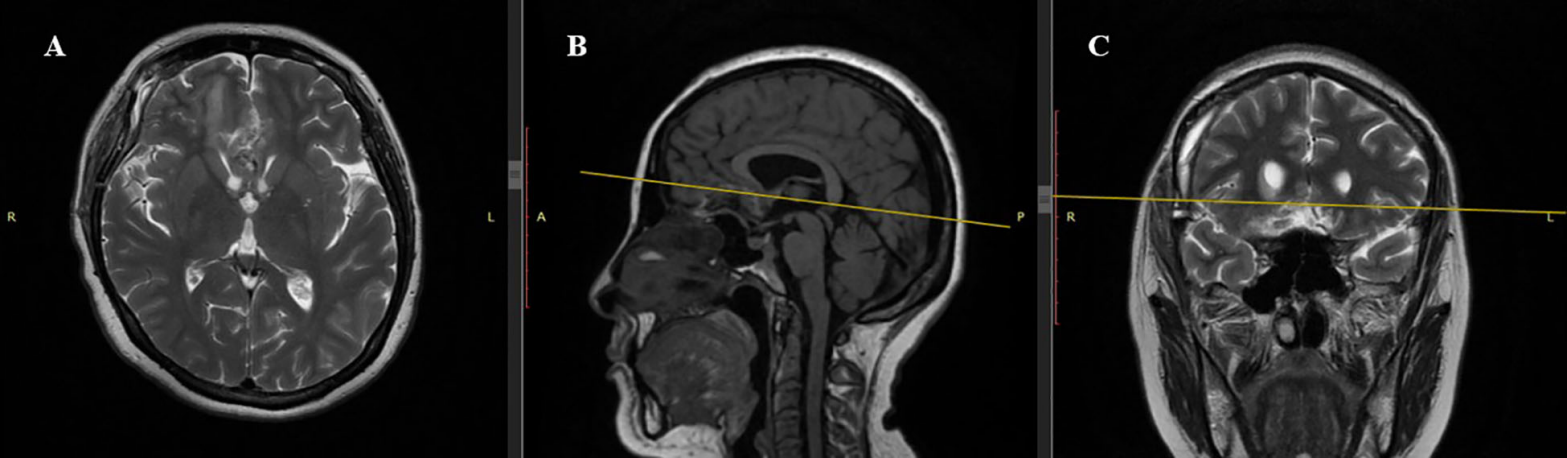
Postoperative MRI Scan (1 month after surgery). **(A)** Axial T2-weighted sequence: Complete resection of the tuberculum sellae meningioma. Frontal lobes show no significant mass effect. **(B)** Sagittal T1-weighted sequence: Hypointense resection cavity with no residual tumor. Midline structures, including the corpus callosum, are realigned without compression of basal cerebral structures. **(C)** Coronal T2-weighted sequence: Hyperintense resection cavity with restored symmetry of frontal lobes and optic chiasm free of compression. No postoperative complications such as hematoma or cerebrospinal fluid accumulation.

Postoperatively, the patient was transferred to the intensive care unit (ICU) under mechanical ventilation with stable hemodynamics. She was successfully extubated within 50 minutes, regained full consciousness, and maintained spontaneous breathing with humidified oxygen. On postoperative day 1, she remained neurologically intact, hemodynamically stable, and reported no pain; diuresis was preserved, and laboratory parameters were within normal limits. On day 2, oral intake was resumed, and the patient mobilized with assistance; no new neurological deficits were observed. On day 3, she was transferred from the ICU to the neurosurgery ward in stable condition. Ophthalmologic examination confirmed stabilization of visual function, with light perception preserved bilaterally and no further deterioration. The neonate, delivered via cesarean section, was a female infant with Apgar scores of 8 and 9 at one and five minutes, respectively, and was admitted to the neonatal intensive care unit for prematurity-related observation. No maternal or neonatal complications occurred. Multidisciplinary follow-up was arranged. The patient was discharged on postoperative 7^th^ day in good general and neurological condition. At the 1-month follow-up, the patient demonstrated stable neurological status with no new deficits. Ophthalmological assessment revealed partial improvement in visual function, with restoration of peripheral vision in the left eye and stable light perception in the right eye. Follow-up MRI confirmed complete macroscopic resection of the lesion, with no radiological signs of residual tumor, recurrence, or procedure-related complications. The imaging findings demonstrated fully resolved mass effect, decompression of the optic apparatus, and restoration of the anterior cranial base anatomy. A detailed radiological assessment is presented in **Figures 3**, **4**.

## Discussion

Meningiomas are the most common benign and nonglial brain tumors, occurring twice as frequently in women (38%) as in men (20%) (7, 8). Among individuals of reproductive age, the incidence ratio of meningiomas in females to males is 3.15:1 and hormonal influences likely contribute to this discrepancy (9). Additionally, according to data from the Central Brain Tumor Registry of the United States, the highest meningioma female-to-male incidence was observed in patients aged 35-54. In this age group, incidence of meningiomas was 3.29 times higher in females than in males (10). A hormonal influence is additionally noted through the association of meningiomas with hormone-dependent malignancies such as breast cancer. Specifically, women previously diagnosed with breast cancer exhibit a 26% higher overall risk of developing meningiomas compared to the general population, with significantly elevated risk in subgroups such as those aged 18–49 years (116% increase) and patients with stage IV breast cancer (139% increase) (11). This may be attributed to the increased availability of diagnostic technologies for women of reproductive age, as well as the potential role of pregnancy-related hormonal changes in accelerating the growth of certain brain tumors. In any case, meningiomas during pregnancy present a clinical challenge. These tumors may affect maternal health and pose potential risks to the fetus.

Pregnancy can significantly impact meningioma growth due to hormonal and hemodynamic changes. Elevated progesterone and estrogen levels in the second and third trimesters can stimulate tumor proliferation via progesterone receptors (12). In addition, increased circulating blood volume and upregulation of vascular endothelial growth factor (VEGF) may enhance tumor vascularization and promote peritumoral edema. The resulting mass effect and edema can produce or exacerbate symptoms such as headache, visual disturbances, and seizures (9). Postpartum, as hormone levels and blood volume normalize, many symptoms can resolve spontaneously. Some research suggests that pregnancy-associated meningioma growth is often reversible and linked more to hemodynamic shifts than true cellular proliferation (10). The role of gonadotropic hormones - luteinizing hormone, follicle-stimulating hormone, and human chorionic gonadotropin - has also been explored. Boyle-Walesh et al. noted that these hormones may indirectly influence tumor growth by modulating hormone receptor expression and interacting with the hypothalamic-pituitary-gonadal axis (13). Although multiple laboratory and clinical studies support the involvement of sex hormones in the growth of meningiomas, the data remain heterogeneous and sometimes contradictory. Overall, current evidence suggests that conditions characterized by pronounced hormonal fluctuations, such as pregnancy, can modulate meningioma behavior, although further research is required to elucidate the exact physiological mechanisms underlying this association.

Peritumoral edema is a key factor in clinical deterioration and, when present, may increase the risk of seizures in late pregnancy and requiring urgent intervention (9). In our case, pregnancy was prolonged to minimize neonatal risks, but rapid vision loss necessitated immediate surgery. Over two months pre-hospitalization, tumor growth accelerated, likely due to hormonal and hemodynamic changes. This highlights the need for careful monitoring, clinical vigilance, and adaptive treatment strategies for pregnant patients with meningiomas. Brain tumor symptoms during pregnancy - headaches, nausea, vomiting, and vision impairment – can be misinterpreted as normal pregnancy-related changes, complicating early diagnosis (14). Our patient initially reported headaches, vision loss, weakness, and fatigue, which could have been mistaken for pregnancy symptoms. However, progressive worsening, culminating in near-total vision loss, prompted timely tumor detection. This underscores the importance of clinical vigilance in pregnant women with atypical and worsening neurological symptoms, as delayed diagnosis often leads to advanced-stage detection and worsened prognosis.

Diagnosing brain tumors during pregnancy is complex due to the need to minimize fetal risks. Non-contrast MRI is the preferred method, and contrast-enhanced imaging is recommended only when benefits outweigh potential risks (15). Gadolinium-based contrast agents cross the placenta, accumulate in fetal tissues, and pose risks to fetal development especially during organogenesis, leading the FDA to advise against their use in the first trimester (16). This limitation often reduces imaging clarity, complicating tumor evaluation. In our case, despite the absence of contrast, standard MRI successfully identified a 4.81×4.85×4.26 cm tuberculum sellae meningioma, demonstrating that non-contrast imaging can still provide critical diagnostic information. However, when a lack of accurate imaging could endanger the mother’s life, gadolinium-based contrast agents may be considered, following a comprehensive discussion of risks and benefits. Computed tomography (CT) is another alternative in emergencies, though it involves radiation exposure. While its 2 mSv dose is significantly lower than the teratogenic threshold (50 mSv), its use remains cautious and situation-dependent (17, 18). In our case, rapid vision deterioration and worsening neurological status demanded urgent imaging, underscoring the importance of balancing diagnostic accuracy with maternal and fetal safety.

There are several important considerations when determining the appropriate management of brain tumors during pregnancy. Various treatment modalities - including surgical resection, conservative management, and adjuvant therapies - must be carefully selected based on tumor type, gestational age, and maternal-fetal risk balance. Surgical removal of brain tumors during pregnancy is indicated when rapid tumor growth or malignancy threatens maternal life. The optimal surgical window is the second trimester (13–28 weeks), when risks of miscarriage (10-15%) and preterm labor (<5%) are lowest. However, in the third trimester, the risk of preterm delivery rises to 20-30%, particularly with aggressive tumors like glioblastomas (18–20). In stable patients, surgery is often delayed until the second trimester (21), but progressive neurological decline, hydrocephalus, or severe edema necessitate urgent intervention (20). Though it is generally recommended to delay surgery to the second trimester (22), our patient’s rapid vision deterioration and risk of optic nerve atrophy necessitated simultaneous tumor resection and cesarean section. For stable first-trimester cases, surgery is typically deferred, and adjuvant radiation therapy may be considered later if indicated depending on tumor type. In unstable patients, surgery is required regardless of gestational age. In the late second and early third trimesters, patients are often monitored unless symptoms worsen, in which case radiation therapy can provide temporary relief for more aggressive tumors until planned cesarean section and neurosurgical intervention (22).

In select cases, when surgical intervention cannot be safely postponed and fetal maturity is deemed sufficient, a combined approach involving simultaneous cesarean delivery and tumor resection may be considered to mitigate the risks associated with sequential procedures and repeated anesthesia exposure. While isolated reports exist describing staged or postponed interventions, simultaneous cesarean section and brain tumor resection remain exceptional and are typically reserved for rapidly progressive or life-threatening conditions. A systematic review by Guerrero-Ocampo et al. identified only seven cases in the literature wherein such simultaneous procedures were performed, demonstrating varied approaches depending on gestational age, tumor location, and clinical urgency (23). These included parasagittal, frontal, and suprasellar meningiomas between 26 and 39 weeks of gestation, with most procedures initiated due to worsening neurological symptoms or fetal distress. Additionally, Guerrero-Ocampo et al. contributed their own case of a 40-year-old patient with a giant frontal meningioma at 28 weeks, successfully managed with emergent decompressive craniectomy and cesarean delivery following intraoperative fetal bradycardia. Beyond this, further reports by Guerrero Ortiz and Chung et al. describe successful outcomes following simultaneous resection of a cerebellopontine angle meningioma and pituitary adenoma, respectively (24, 25). Simultaneous cesarean section and neurosurgical tumor resection may offer several clinical advantages in such scenarios. First, performing both procedures in a single session avoids the risks associated with repeated general anesthesia and minimizes perioperative hemodynamic instability — a crucial consideration in patients with evolving mass effect and impaired intracranial compliance (23). Second, this approach eliminates the dangers associated with vaginal delivery in patients with intracranial lesions, particularly those at risk for herniation due to increased intracranial pressure during labor (26). Third, delivering the fetus before neurosurgical manipulation prevents fetal exposure to intraoperative agents such as mannitol, hyperventilation, or high-dose corticosteroids, which may adversely affect fetal perfusion and development if surgery is performed antenatally without cesarean section (25, 26). These factors underscore the need for individualized multidisciplinary planning in complex neurosurgical-obstetric cases. Our current case, involving a 35-year-old woman at 38 weeks of gestation with a giant tuberculum sellae meningioma (WHO Grade 2), stands out due to the full-term status of the pregnancy and the specific surgical approach utilized. To our knowledge, this is the only reported case combining a pterional craniotomy with cesarean section for this tumor location. This simultaneous intervention enabled immediate restoration of the patient’s visual function and the safe delivery of a healthy infant, minimizing the risks associated with delayed surgery or repeated anesthesia. Notably, the tumor’s atypical histology (Grade II) highlights the importance of long-term neuro-oncological follow-up, adding complexity to both perioperative and post-discharge planning.

In the management of brain tumors during pregnancy, symptom control is often an essential component of treatment, particularly in cases where immediate surgical intervention is not required. Corticosteroids, most notably dexamethasone, are frequently used to reduce peritumoral edema and mitigate neurological symptoms such as headache, nausea, and visual disturbances. Their mechanism targets vasogenic edema associated with the tumor, rather than direct tumoricidal activity. As reported by Arias A. et al., corticosteroid therapy can effectively alleviate symptoms associated with tumor-related mass effect in clinically stable patients (21). The systematic review by Kemp et al. outlines standard antenatal corticosteroid regimens - such as betamethasone administered as two 12 mg intramuscular injections 24 hours apart, or dexamethasone given as four 6 mg doses every 12 hours - as commonly accepted clinical protocols. However, as the authors emphasize, these dosing strategies are not grounded in randomized dose-finding trials but are instead derived from clinical convention, lacking robust pharmacokinetic validation (27). In cases of rapid symptom progression or impending neurological compromise, however, surgical resection becomes the preferred approach. While corticosteroid therapy may offer symptomatic relief in selected non-emergent cases, its use during pregnancy must be carefully evaluated due to emerging evidence of potential adverse fetal outcomes, including impaired growth and long-term neurodevelopmental effects (28). These concerns highlight the need for further research in large cohorts with long-term follow-up to better establish the safety profile of corticosteroid use in pregnant patients with brain tumors.

The prognosis for pregnant patients with brain tumors depends largely on the tumor type and stage. According to a large-scale retrospective cohort study by Terry et al., which analyzed 19,750,702 pregnancy-related hospitalizations in the United States, the prognosis varies significantly between malignant and benign brain tumors. Among 379 cases of malignant brain tumors, these neoplasms were strongly associated with adverse maternal and fetal outcomes, including a substantial risk of maternal mortality (<10%), preterm delivery (19.4%), and intrauterine fetal demise (<10%). Conversely, benign brain tumors, documented in 437 cases, while posing a lower threat to maternal survival, were associated with a markedly increased incidence of cesarean section (49.4%) and preterm labor (15.9%) (29). This finding is particularly noteworthy, as it suggests that even non-malignant intracranial tumors can substantially impact obstetric decision-making. The elevated rates of surgical delivery and preterm birth may be attributed to mass effect, increased intracranial pressure, and concerns over exacerbation of neurological symptoms during labor. As a result, clinicians often opt for early or elective cesarean section as a precautionary measure. Advances in obstetric care have significantly reduced maternal mortality associated with pregnancy, but non-obstetric complications such as hypertension and disseminated intravascular coagulation remain major concerns, especially in patients with brain tumors (30). These conditions require individualized, multidisciplinary management strategies that account for both the physiological adaptations of pregnancy and the neurological risks posed by intracranial neoplasms.

A multidisciplinary approach played a pivotal role in the successful management of this case. The coordinated efforts of neurosurgeons, obstetricians, anesthesiologists, neonatologists, and neuro-oncologists ensured timely decision-making, intraoperative safety, and postoperative continuity of care. Of particular importance was the anesthesiology team, which adapted intraoperative parameters to balance cerebral protection with fetal oxygenation and hemodynamic stability. This strategy not only minimized risks for both mother and child but also maximized functional recovery. Contemporary literature supports the use of multidisciplinary protocols in managing brain tumors during pregnancy. For instance, Hamade et al. emphasized the crucial role of coordinated neuro-oncological care in pregnant patients with gliomas, particularly to avoid delays in life-saving treatment and to mitigate fetal risks through individualized planning (31). Similarly, Martin et al. demonstrated how integrated decision-making across specialties led to a successful outcome in a patient with a meningoendothelial meningioma during pregnancy (32). Accordingly, our case may serve as a model for future management algorithms in similarly complex clinical scenarios, reinforcing the necessity of institutional preparedness, protocol development, and interdisciplinary communication when addressing rare but high-stakes neurosurgical emergencies in obstetric patients.

## Conclusion

The management of intracranial tumors during pregnancy requires an individualized and meticulously planned multidisciplinary approach based on a comprehensive assessment of tumor-related clinical manifestations, physiological changes during pregnancy, and potential risks to both the mother and the fetus. In the presented clinical case, timely diagnosis and the implementation of a strategically designed treatment plan allowed for the minimization of complications and the achievement of an optimal outcome: successful removal of a giant meningioma, restoration of the patient’s visual function, and safe delivery. The simultaneous neurosurgical intervention and cesarean section demonstrate the effectiveness of a combined surgical approach in critical cases where delaying treatment could lead to irreversible consequences. This case highlights the importance of multidisciplinary collaboration among neurosurgeons, obstetricians, anesthesiologists, neonatologists, and oncologists, as well as the necessity of developing a clear management algorithm for pregnant patients with intracranial tumors. Such an algorithm should include early diagnosis and dynamic monitoring, coordination among specialists, an individualized approach to surgical intervention based on gestational age, anesthetic management with risk minimization for the fetus, and postoperative follow-up with oncological support if necessary. The development of standardized clinical guidelines will help optimize treatment strategies, enhance the safety and effectiveness of patient management, and ensure the most favorable perinatal and neurological outcomes.

## Data Availability

The original contributions presented in the study are included in the article/**Supplementary Material**. Further inquiries can be directed to the corresponding author.
