# Supplementary figures and images for: Simultaneous surgical management of a giant tuberculum sellae meningioma and pregnancy-related complications: a case report and literature review
Source: Front Oncol. 2025 Jun 4;15:1576797. doi: 10.3389/fonc.2025.1576797 (PMC12173878; doi:10.3389/fonc.2025.1576797)

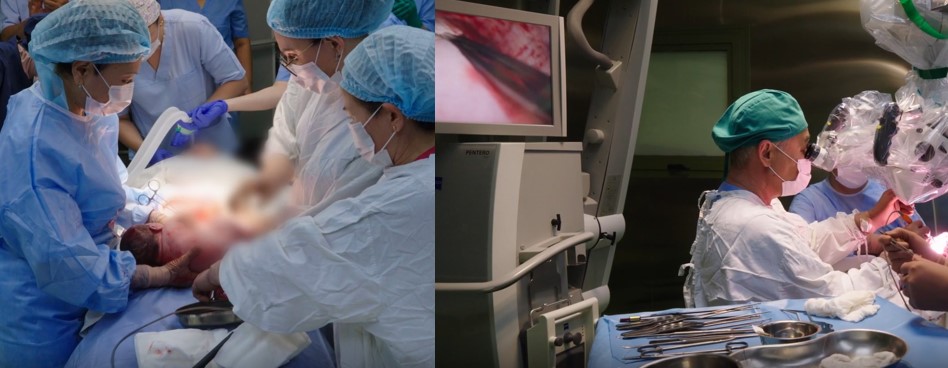

Supplement: Supplementary file 1 [file Image1.jpeg]
